# Drug Delivery Systems of Betulin and Its Derivatives: An Overview

**DOI:** 10.3390/biomedicines12061168

**Published:** 2024-05-24

**Authors:** Bartosz Jaroszewski, Katarzyna Jelonek, Janusz Kasperczyk

**Affiliations:** 1 Department of Biopharmacy, Faculty of Pharmaceutical Sciences in Sosnowiec, Medical University of Silesia, Jedności 8, 41-200 Sosnowiec, Poland; d201131@365.sum.edu.pl; 2 Centre of Polymer and Carbon Materials, Polish Academy of Sciences, Curie-Skłodowska 34 St., 41-819 Zabrze, Poland

**Keywords:** betulin, betulin derivative, drug delivery system, nanoparticles, nanocarriers, microparticles, natural drugs, anticancer drugs

## Abstract

Natural origin products are regarded as promising for the development of new therapeutic therapies with improved effectiveness, biocompatibility, reduced side effects, and low cost of production. Betulin (BE) is very promising due to its wide range of pharmacological activities, including its anticancer, antioxidant, and antimicrobial properties. However, despite advancements in the use of triterpenes for clinical purposes, there are still some obstacles that hinder their full potential, such as their hydrophobicity, low solubility, and poor bioavailability. To address these concerns, new BE derivatives have been synthesized. Moreover, drug delivery systems have emerged as a promising solution to overcome the barriers faced in the clinical application of natural products. The aim of this manuscript is to summarize the recent achievements in the field of delivery systems of BE and its derivatives. This review also presents the BE derivatives mostly considered for medical applications. The electronic databases of scientific publications were searched for the most interesting achievements in the last ten years. Thus far, it is mostly nanoparticles (NPs) that have been considered for the delivery of betulin and its derivatives, including organic NPs (e.g., micelles, conjugates, liposomes, cyclodextrins, protein NPs), inorganic NPs (carbon nanotubes, gold NPs, silver), and complex/hybrid and miscellaneous nanoparticulate systems. However, there are also examples of microparticles, gel-based systems, suspensions, emulsions, and scaffolds, which seem promising for the delivery of BE and its derivatives.

## 1. Introduction

The interest in natural products as potential candidates for clinical prevention, diagnosis, and treatment is growing due to their proven significant curative effects. Compared to synthetic drugs, natural products not only have diverse structures but also exhibit a range of biological activities against different disease states and molecular targets, making them attractive for development in the field of medicine [1]. The use of natural plant-derived compounds has been considered as an interesting aspect for medical and pharmaceutical applications. Researchers have focused on a certain group of triterpenes derived from betulin (BE) since the 1990s [2]. BE, chemically known as lup-20(29)-ene-3β,28-diol, is a naturally occurring triterpene characterized by a five-membered ring and an isopropylidene group. Many species of plants contain BE and have been used to search for new compounds and to synthesize naturally occurring derivatives of BE. New methods of synthesis and extraction have been developed, focused on improving the efficiency of the process and reducing the cost of production, e.g., by using waste from the processing of wood material [3]. Much effort has also been geared toward modifying the original carbon skeleton of BE in order to increase its solubility, enhance its pharmacokinetics, and broaden its range of biological activities. Therefore, recent works have focused on the synthesis of new molecules such as betulinic acid, betulone, and betulonic acids, and other BE derivatives [4]. Most studies related to the assessment of the possibilities inherent in BE and its derivatives have provided promising results both in vitro and in vivo [5,6,7,8].

Despite advancements in the use of natural products for clinical purposes, there are still some obstacles that hinder their full potential, e.g., their limited solubility and stability when administered orally or their short duration of effectiveness. To address these concerns, drug delivery systems have emerged as a promising solution to overcome the barriers faced in the clinical application of natural products [1]. Moreover, there is a need for the development of delivery systems of BE and its new derivatives due to the characteristics of triterpenes, such as hydrophobicity, low solubility, and the resulting poor bioavailability [5,6].

The aim of this manuscript is to summarize the recent achievements in the field of delivery systems of betulin and its derivatives. This review also presents the most interesting betulin derivatives for medical applications. The subject is important because the newly developed carriers of BE may increase the biological potential of the active agents and result in faster clinical translation. There have been many review papers that focused on the extraction process of BE [3] production of its derivatives [9], or presenting the activity and mechanisms of action of BE or its derivatives [6,8,10,11,12,13,14,15,16]. However, analysis of the delivery systems of BE and its derivatives has not been published so far.

## 2. Methodology

Electronic databases of scientific publications (i.e., PubMed, Science Direct, Web of Science, Google search) were searched. This review presents the most interesting achievements in the subject of betulin derivatives for medical applications and in the field of delivery systems of betulin and its derivatives in the last ten years.

## 3. Characteristics of Betulin and Betulinic Acid and Their Pharmaceutical Potential

### 3.1. Betulin

**Figure 1:**
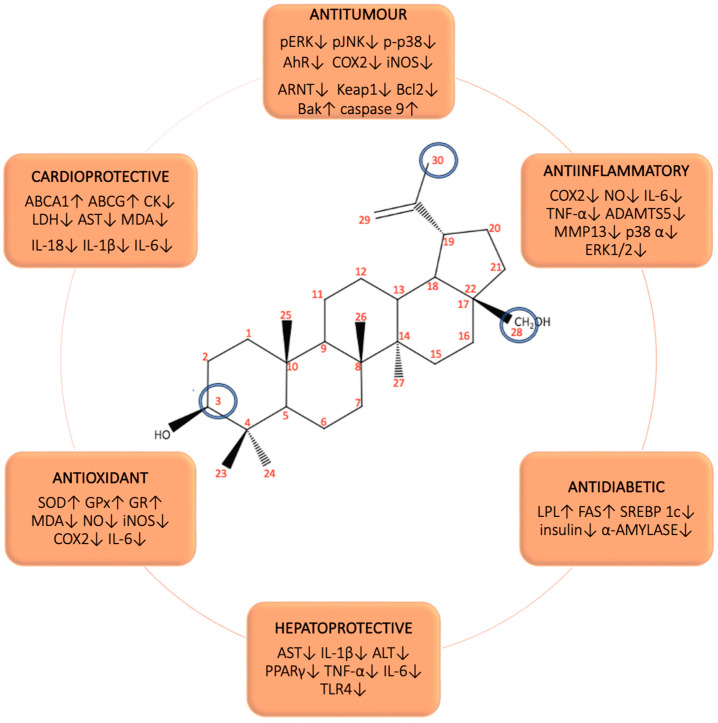
A schematic presentation of the biological effects of betulin and its chemical structure. The C-3, C-28, and C-30 groups considered for chemical modification are marked.

Betulin (BE) (Figure 1), Lup-20(29)-ene-3β,28-diol, a pentacyclic triterpene, can be found in the bark of large quantities of plant species. BE, together with its derivatives, is an object of many research studies, which are devoted to assessing their multidirectional activity. The advantages of BE include its low toxicity, good availability, and low cost of synthesis. Furthermore, the structure is susceptible to modification, which further enhances its potential of application. However, BE is characterized by poor bioavailability, which is limited by its hydrophobic properties [17].

A rich source of BE is plants of the *Betulaceae* family, including *Betula alba*, *B. pendula*, *B. pubescens*, and *B. platyphylla* [4]. The white color of birch bark is caused by BE, which occurs in spring as clusters of crystals in thin-walled, large cells [6]. Plants synthesize this ingredient in order to protect against all harmful environmental factors, such as bacteria, fungi, viruses, insects, or radiation [3]. Depending on factors such as tree species, tree age, weather conditions, or geographic location, the BE content in dry weight can range from 10% to 45%. BE is also present in much smaller quantities in the roots and leaves. Birch bark extracts contain between 70% and 80% of BE [6]. Therefore, birch bark can be considered an economical raw material for obtaining triterpene compounds, such as BE [18].

The most common way to obtain BE is extraction from birch bark with the use of organic solvents like methanol, dichloromethane, chloroform, and acetone followed by crystallization of the extract. However, other triterpene compounds may pass into the extract, so, usually, purification is required to obtain BE [3,19], which is costly and time-consuming [3,19]. Therefore, methods improving the extraction process of BE from birch bark have been developed. Such methods include the extraction of BE from an alkaline environment in a microwave field, which allowed an increase in the extraction rate by 15–20 times and significantly accelerated the process in comparison to the standard extraction procedure [20]. Additionally, the advantageous method of BE extraction with the use of aqueous propan-2-ol has been developed, which does not require crystallization [21]. A five-stage purification process has been studied to increase the purity of BE with a yield of about 20% [22]. The method developed by Birken Gmbh to obtain BE from birch cork cambium, which is waste from the pulp mill, allows BE to be obtained with a purity of 80–90% and has been protected by a patent (EP 1 758 555 B1) [16].

BE has gained the interest of researchers due to its wide range of biological effects. The anticancer potential of BE is considered to be extremely valuable due to the need to develop a compound with high activity and few side effects [4]. Reports regarding the sensitivity of cancer cells to BE involve various cell lines, such as cervical cancer (HeLa), epidermoid cancer (A431), breast cancer (MCF-7), stomach cancer (SGC7901), lung cancer (A549), and liver cancer (HepG2) [23,24,25]. Besides its strictly anticancer effect, the anti-inflammatory effect of BE is very important. The ability to reduce inflammation and influence pro-inflammatory cytokines, signaling pathways, and the reduction in reactive oxygen species has been confirmed in cellular and animal models [4,11,25,26]. BE is also described as an agent against pathogens causing infectious diseases such as fungi, bacteria, parasites, and viruses, including HIV [4,6,25]. Furthermore, the efficacy of BE in the management of cardiovascular diseases, liver diseases, diabetes, and wounds has been noted [4,6,25]. Figure 1 presents a summary of the parameters influenced by BE.

Apart from preclinical studies, several clinical trials have also been conducted on BE, ranging from anti-hepatitis activity to wound-healing effects [4].

### 3.2. Betulinic Acid

Over the past few years, numerous BE derivatives, such as betulinic acid (BA), betulonic acid (BoA), and 23-hydroxybetulinic acid (HBA), have been synthesized, which characterized targeted, specific action and better bioavailability in comparison to the original compound [6,27].

**Figure 2:**
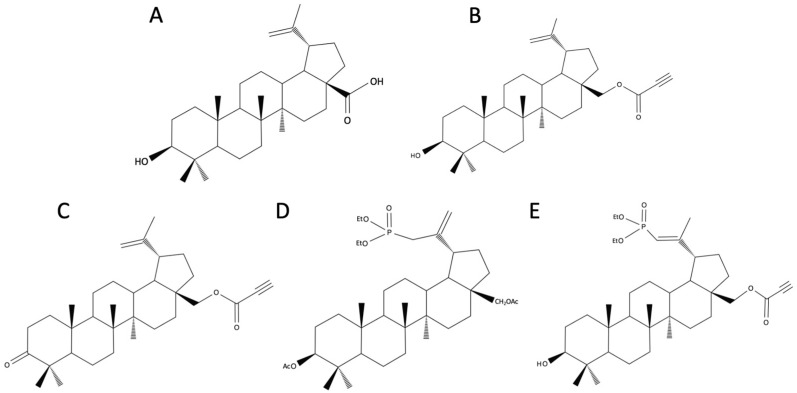
Chemical structure of betulinic acid (**A**), 28-O-propynoylbetulin (**B**), 28-O-propynoylbetulone (**C**), 3β,28-diacetoxy-30-diethoxyphosphoryl-lup-20(29)-ene (**D**), and 29-diethoxyphosphoryl-28-propynoyloxy-lup-20E(29)-en-3-ol (**E**).

BA (Figure 2A), (3β)-3-Hydroxy-lup-20(29)-en-28-oic acid, occurs in various plant species (mainly *Betula* sp.) and some fungi. BA and BE differ structurally in the presence of the carboxylic group instead of hydroxymethyl at C17 [6,16,28]. BA can be obtained as a by-product during BE purification or by birch bark extraction [22]. However, the concentration of BA in birch bark is 0.002–2%, which is a limiting factor of the extraction [29]. Multiple methods for the synthesis of BA from BE have been described, but they are expensive and time-consuming for commercial use [29].

Due to the limitations of obtaining BA directly from plant material and the transformation of BE to BA by chemical synthesis, there are several alternative methods that are catalyzed by fungi. Several strains of fungi were examined and utilized to transform BE into BA, including *Armillaria luteo-virens Sacc ZJUQH100-6* [30,31], *Aspergillus foetidus*, *Aspergillus oryzae* [31], and *Cunninghamella blakesleeana* [32]. In order to fully utilize the potential of these fungi strains, additional research is necessary to identify the conditions that enhance the efficacy of the biotransformation process and to identify chemical factors that can serve as biocatalysts of this process. Another strain that has been tested is *Inonotus obliquus* [33], which has garnered attention due to the presence of over 10 triterpenoids, including BA. Efforts have also been devoted to stimulation of the microorganisms to enhance the concentration of BA and to discover factors that may enhance its production. These factors include oleic acid, fungal elicitor, and BE [33]. Methods for the synthesis of BA from BE using transgenic yeast have also been studied [29,34]. Wu et al. described a biotransformation of BE to BA using a microsomal protein fraction from modified *Saccharomyces cerevisiae*. The cell-free production design allows not only intensification of the production of BA, but also elimination of the cytotoxic effect of BE on microbes. However, despite the fact that the obtained results are promising, they require further research in order to optimize and increase the efficiency of the process [34]. The possibility of obtaining BA from plant tissue culture has also been reported [29].

BA presents a significant potential for medical applications, and, similar to BE, it possesses anticancer, antiviral, antimicrobial, and anti-inflammatory properties [10,13,14,15,35]. BA may induce cell death in vitro in many types of tumors, including pancreatic, breast, hepatoma, glioma, leukemia, ovarian, cervix, prostate, lung, and colorectal cancers [36]. It inhibits topoisomerase, induces apoptosis by caspase 3 activation, and causes mitochondrial membrane damage [37]. BA also stimulates reactive oxygen species (ROSs) and impairs antioxidant systems in cancer cells [38]. The cytotoxic effects of the compound against healthy cells are minimal, rendering BA a promising potential anticancer drug [37]. In addition, there are reports of its anti-inflammatory effect [8], ability to inhibit HIV-1 [7,39], and antimalarial activity [12]. BA has also been tested as an antidiabetic factor [40]. In vitro and in vivo studies confirm the beneficial effect of BA on glucose metabolism in the body. In addition to reducing hyperglycemia, BA may also protect from some complications of diabetes. BA is considered to have antidepressant or hepatoprotective and renal protective properties [6,29,41].

## 4. Characteristics of BE Derivatives and Their Biological Properties

As presented in Section 3, BE and BA are interesting biologically attractive natural parent molecules with a high safety profile that can easily undergo a variety of structural modifications. Modifying carbons at positions 3, 28, and 29 results in the creation of new derivatives with great application potential (Figure 1).

### 4.1. Acetylenic Derivatives of Betulin and Betulone

The advancement of research into the enhancement of anticancer potential by substituting the C-28 position with alkane, alkene, and alkynyl groups resulted in the discovery of numerous BE and betulone derivatives [42].

A new series of BE derivatives have been synthesized that contain one or two pharmacophores bearing an acetylenic and carbonyl function at the C-3 and/or C-28 positions. The new compounds were tested in vitro for their antiproliferative activity against human colorectal (SW707), leukemia (CCRF/CEM), and breast (T47D) cancer cell lines, a murine leukemia cell line (P388), and normal fibroblasts (Balb3T3). It was found that the BE derivatives that possess a carbonyl group at the C-28 position directly bonded to the triple bond of an ethynyl substituent, showed strong cytotoxic effects against CCRF/CEM and P388 cancer cells. Additionally, the free hydroxyl group in the C-3 position is an essential element of the molecule, determining the pharmacological activity of the tested BE acetylenic derivatives. 28-O-propynoylbetulin (Figure 2B) even showed 500 times higher cytotoxicity than BE and 100 times higher cytotoxicity than cisplatin against CCRF/CEM cells [43].

Additionally, 28-O-propynoylbetulone (Figure 2C) exhibits high anticancer activity in vitro against HL-60 leukemia cell lines, which is comparable with cisplatin and surpasses the cytotoxic activity of BE. The research also discussed the relationship between the structure and activity of monoesters, highlighting the importance of alkyl groups at the C-28 position and oxidation at the C-3 position for the anticancer activity of BE derivatives [44].

The effect of acetylenic synthetic BE derivatives on glioma cells (T98G and C6) was evaluated. The reduced viability and inhibited proliferation of glioma cells was observed and the effect was higher than the routinely used chemotherapeutic drugs, i.e., cisplatin and temozolomide [45].

### 4.2. Betulin Phosphonates

Chrobak et al. synthesized a BE derivative, 3β,28-diacetoxy-30-diethoxyphosphoryl-lup-20(29)-ene (Figure 2D), containing a phosphonate group located at the C-30 position (Figure 1). Derivatives containing a phosphonate group located at the C-29 position and acetylenic derivatives were also synthesized. The obtained compounds were tested in vitro for their antiproliferative activity against human breast cancer (T47D), glioblastoma (SNB-19), and melanoma (C32) cell lines. The study showed that the introduction of a phosphonate group to the isopropenyl moiety of BE led to an increase in activity against the T47D and C32 cell lines. Moreover, the derivatives containing this substituent at C29 exhibited higher activity than in position C30 against T47D and SNB-19 cells. The cytotoxic activity of 29-diethoxyphosphoryl-28-propynoyloxy-lup-20E(29)-en-3-ol (Figure 2E) against cancer cells was higher than the effect caused by BE and cisplatin, which shows its anticancer potential [42].

### 4.3. Betulin Phosphates

**Figure 3:**
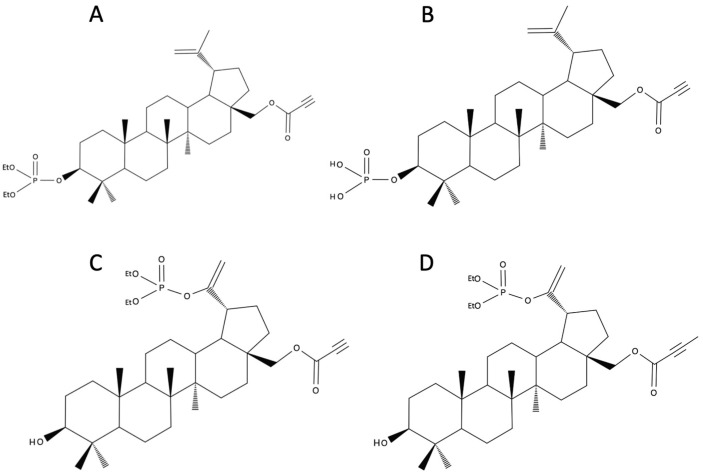
Chemical structure of 3-diethoxyphosphoryl-28-propynoylbetulin (**A**), 3-dihydroxyphosphoryl-28-propynoylbetulin (**B**), 30-diethoxyphosphoryloxy-28-O-propynoylbetulin (**C**), and 28-(2-Butynoyl)-30-diethoxyphosphoryloxybetulin (**D**).

The addition of a phosphate group to the BE molecule may solve the problems with poor solubility and bioavailability but also expand the range of pharmacological treatments. Therefore, several phosphate derivatives of BE and BA have been synthesized [46]. 3-diethoxyphosphoryl-28-propynoylbetulin (Figure 3A) exhibited cytotoxic activity against melanoma cells and 3-dihydroxyphosphoryl-28-propynoylbetulin (Figure 3B) against leukemia cells. The molecular modelling enabled identification of the peroxisome proliferator-activated receptor PPARγ as a molecular target that defines the mechanism of anticancer activity of the tested compounds [46]. It was attempted to answer the question as to whether the replacement of phosphonate with a phosphate group in the isopropenyl substituent affects the activity of BE alkynyl derivatives. The cytotoxic activities of two new derivatives, 30-diethoxyphosphoryloxy-28-propynoylbetulin (Figure 3C) and 28-(2-butynoyl)-30-diethoxyphosphoryloxybetulin (Figure 3D), against breast cancer (T47D, MDA-MB-231), melanoma (C-32), and glioblastoma (SNB-19) cell lines were compared with BE and cisplatin. Breast cancer (T47D, MDA-MB-231) cell lines exhibited a lower sensitivity to the synthesized derivatives compared to melanoma and glioblastoma cell lines. When comparing the activity of BE alkynyl phosphate with BE alkynyl phosphonate, an increase in activity can be observed in certain instances. However, comparison of the most potent compounds from both groups does not show significant difference in their efficacy against glioblastoma and melanoma cell lines. Furthermore, BE alkynyl phosphate exhibits a weaker effect on breast cancer lines [47].

Additionally, 30-diethoxyphosphoryloxy-28-O-propynoylbetulin (Figure 3C) was also tested in vitro against SK-BR-3 and MCF-7 breast cancer lines. In both cell lines, it demonstrated a significantly higher activity compared to the parent compound (BE). The inhibition of cell growth correlated with an increased expression of the p21WAF1/Cip1 gene. The decrease in cell viability was preceded by changes in ROS levels and a loss of mitochondrial potential. DNA fragmentation and activation of caspase-3 (in SK-BR-3 cells) indicated the possibility of triggering apoptotic mechanisms. However, cells treated with 30-diethoxyphosphoryloxy-28-propynoylbetulin did not exhibit the nuclear morphology characteristic of apoptosis, such as chromatin condensation and karyorrhexis. 30-diethoxyphosphoryloxy-28-propynoylbetulin caused necrotic-like regulated cell death, which was probably induced by the rapid disruption of mitochondrial function and deficiency of energy [48].

### 4.4. Triazole Hybrids of Betulin and Betulinic Acid

**Figure 4:**
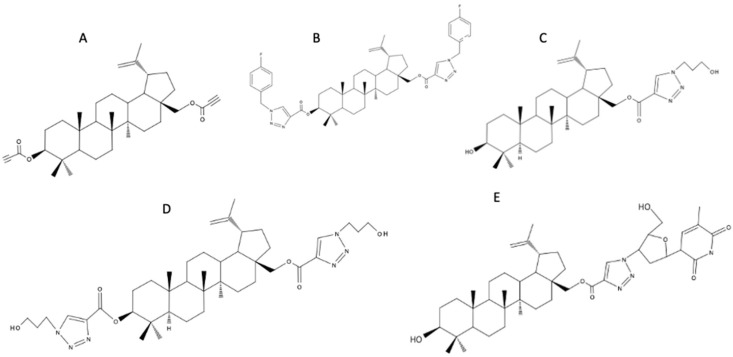
Chemical structure of 3,28-O,O′-di(propynoyl)betulin (**A**), 3,28-O,O′-di[1-(4-fluorobenzyl-1H-1,2,3-triazol-4-yl) carbonyl]betulin (**B**), 28-O-[1-(3-hydroxypropyl)-1H-1,2,3-triazol-4-yl]carbonylbetulin (**C**), 3,28-O,O′-di[1-(3-hydroxypropyl-1H-1,2,3-triazol-4-yl)carbonyl]betulin (**D**), and 28-O-[1-(3′-deoxythymidine-5′-yl)-1H-1,2,3-triazol-4-yl]carbonylbetulin (**E**).

BE derivatives containing a 1,2,3-triazole ring possess a wide spectrum of biological properties, including antiviral, anticancer, and antibacterial activity. A series of novel triazoles were obtained by the 1,3-dipolar cycloaddition reaction between the alkyne derivatives of BE and organic azides. Using 28-O-propynoylbetulin and 3,28-O,O′-di(propynoyl)betulin (Figure 4A), triazole BE derivatives were synthesized. 3,28-O,O′-Di[1-(4-fluorobenzyl-1H-1,2,3-triazol-4-yl) carbonyl]betulin (Figure 4B) demonstrated anticancer activity superior to that of cisplatin against breast cancer lines. 28-O-[1-(3-Hydroxypropyl)-1H-1,2,3-triazol-4-yl]carbonylbetulin (Figure 4C) and 3,28-O,O′-di[1-(3-hydroxypropyl-1H-1,2,3-triazol-4-yl)carbonyl]betulin (Figure 4D) showed greater antiviral activity than BE against ECBO (strain LCR-4) in lung carcinoma cells (A549). 28-O-[1-(3′-deoxythymidine-5′-yl)-1H-1,2,3-triazol-4-yl]carbonylbetulin (Figure 4E) exhibited antibacterial activity against *Klebsiella pneumoniae* and *Escherichia coli* [49].

**Figure 5:**
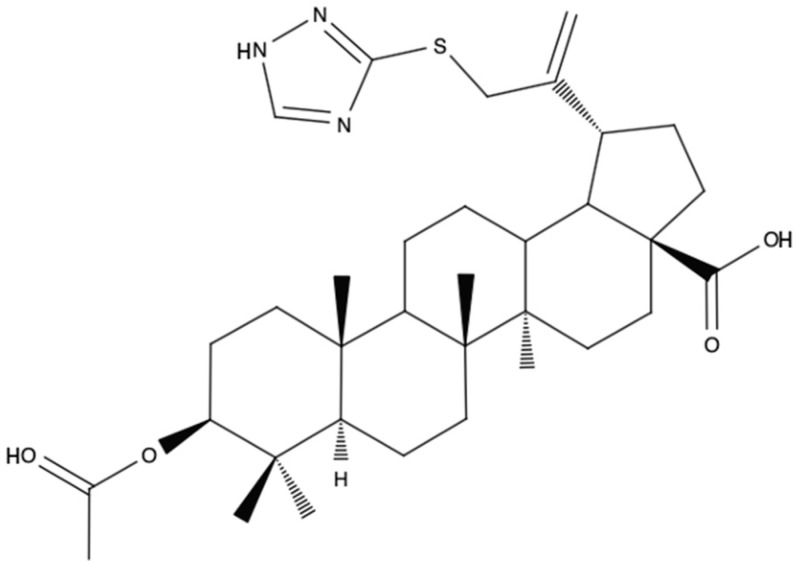
Chemical structure of 3β-O-Acetyl-30-(1H-1,2,4-triazole-3-ylsulfanyl)-betulinic acid.

Furthermore, conjugates of moieties based on a triazole ring with BA have been described. Among the synthesized triazole derivatives of BA, 3β-O-acetyl-30-(1H-1,2,4-triazole-3-ylsulfanyl)-betulinic acid (Figure 5) was selected for analysis of its cytotoxic activity against melanoma cells. The inhibition of the proliferation of melanoma cells (RPMI-7951) was slightly higher than the effect caused by the free forms of BA or 1,2,4-triazole-3-thiol. The tested derivative showed selectivity, because at concentrations of 2 μM and 10 μM, it did not have a toxic effect against the normal cell line (HaCaT). A possible mechanism of action of the derivative, which involves inducing mitochondria-dependent cell apoptosis, was also confirmed [50].

**Figure 6:**
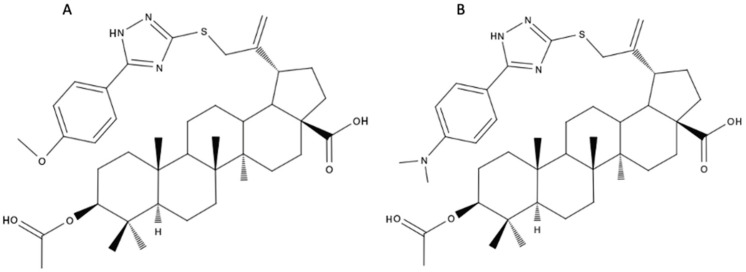
Chemical structure of 3β-O-acetyl-30-[5-(4-methoxyphenyl)-1H-1,2,4-triazol-3-yl)-sulfanyl]-betulinic acid (**A**) and 3β-O-acetyl-30-{5-[4-(dimethylamino)phenyl]-1H-1,2,4-triazol-3-yl)sulfanyl}-betulinic acid (**B**).

The introduction of the triazole moiety was not solely intended to enhance solubility and bioavailability. It was also suggested that the attachment of a substituent with a triazole ring, which is connected to the C-30 carbon on one side and has an additional aromatic moiety at the opposite end, could potentially serve as a novel pharmacophore. It is also worth noting that the substitution of the C-3 carbon affects bioavailability and the attachment of a small acetate molecule may improve penetration of the molecule through cell membranes [51]. Finally, two aryl-substituted 1,2,4-triazole derivatives of BA were selected: 3β-O-acetyl-30-[5-(4-methoxyphenyl)-1H-1,2,4-triazol-3-yl)-sulfanyl]-betulinic acid (Figure 6A) and 3β-O-acetyl-30-{5-[4-(dimethylamino)phenyl]-1H-1,2,4-triazol-3-yl)sulfanyl}-betulinic acid (Figure 6B). These derivatives showed higher toxicities against melanoma cell line (IC50s of 8.8 μM and 20.7 μM, respectively) than the parent BA molecule and induced mitochondria-dependent cell apoptosis [52].

### 4.5. Betulin-1,4-quinone Hybrids

**Figure 7:**
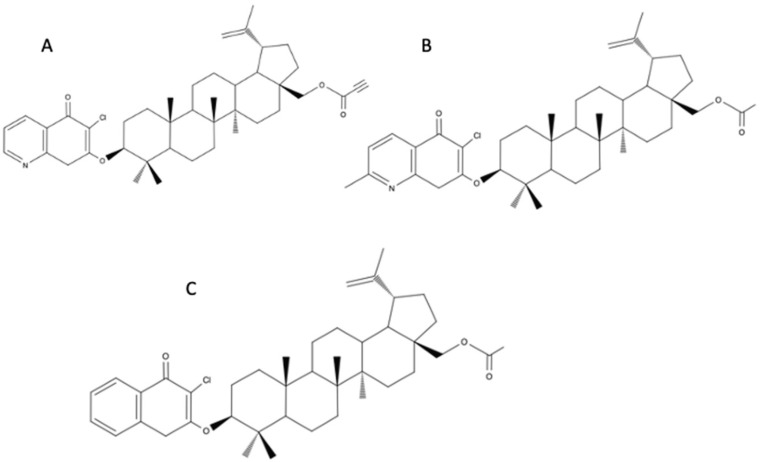
Chemical structure of 6-chloro-7-(28-propynoyl-3-betulinyloxy)-5,8-quinolinedione (**A**), 7-(28-acetyl-3-betulinyloxy)-6-chloro-2-methyl-5,8-quinolinedione (**B**), and 3-(28-acetyl-3-betulinyloxy)-2-chloro-1,4-naphthoquinolinedione (**C**).

The combination of BE with 1,4-quinone resulted in the production of derivatives with high cytostatic activity and greater bioavailability compared to BE. These compounds exhibited a higher activity against various cancer cell lines (lung cancer (A549), breast cancer (MCF-7), and melanoma (C-32) cells) with an increased level of the NAD[P]H-quinone oxidoreductase (NQO1) protein. The selected hybrids were also tested for the transcriptional activity of the gene encoding a proliferation marker (H3 histone), a cell cycle regulator (p53 and p21), and an apoptosis pathway (BCL-2 and BAX). The tested compounds caused a mitochondrial apoptosis pathway in A549 and MCF-7 cell lines [53]. Additional molecular docking studies of selected derivatives provided insight into the activity of new derivatives: 6-chloro-7-(28-propynoyl-3-betulinyloxy)-5,8-quinolinedione (Figure 7A), 7-(28-acetyl-3-betulinyloxy)-6-chloro-2-methyl-5,8-quinolinedione (Figure 7B), and 3-(28-acetyl-3-betulinyloxy)-2-chloro-1,4-naphthoquinolinedione (Figure 7C). The structure–activity relationship showed that biological activity was affected by the type of the 1,4-quinone moiety. Furthermore, it was determined that an interaction of the 1,4-quinone fragment with the hydrophobic matrix near Tyr128, Phe178, Trp105, and the FAD cofactor at the active site may explain the increase in TP53 gene expression [53].

### 4.6. Triphenylphosphonium Analogues of Betulin and Betulinic Acid C3- and C28-Functionalized Derivatives

**Figure 8:**
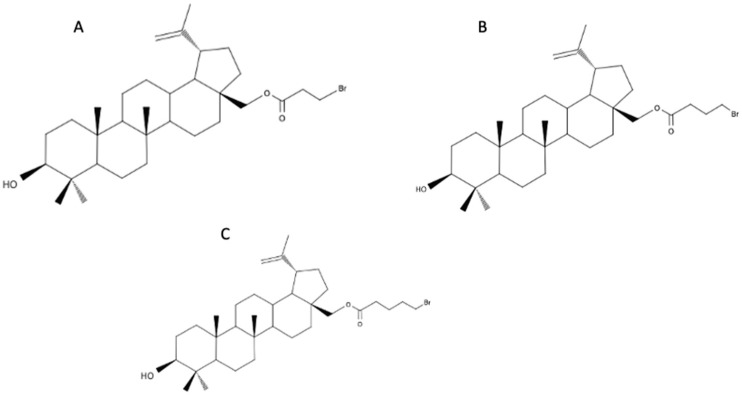
Chemical structure of 3β-Hydroxylup-20(29)-en-28-yl 3-bromopropanoate (**A**), 3β-Hydroxylup-20(29)-en-28-yl 4-bromobutanoate (**B**), and 3β-Hydroxylup-20(29)-en-28-yl 5-bromopentanoate (**C**).

The Steglich esterification process of BE with ω-bromoalkanoic acids leads to the formation of mono- and diesters. Numerous experiments were conducted to evaluate the antimicrobial (bacteriostatic and fungistatic) activity of 3β-hydroxylup-20(29)-en-28-yl 3-bromopropanoate (Figure 8A), 3β-hydroxylup-20(29)-en-28-yl 4-bromobutanoate (Figure 8B), and 3β-hydroxylup-20(29)-en-28-yl 5-bromopentanoate (Figure 8C) against the Gram-positive, Gram-negative strains, and fungal species. Unfortunately, the compounds at the tested concentrations did not demonstrate antimicrobial activity [54].

### 4.7. Betulin Dipropionate

**Figure 9:**
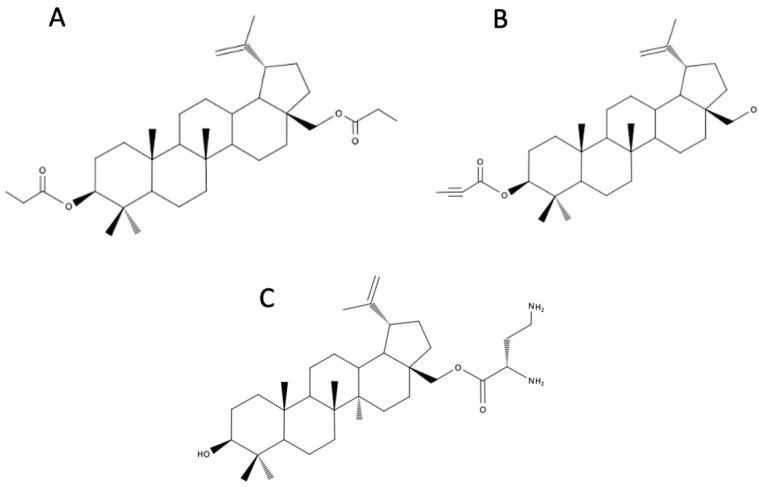
Chemical structure of 3β,28-di-O-propionyl-lup-20(29)-lupene (**A**), 3-(2-butynoyl)botulin (**B**), betulin-dab-NH_2_ (**C**).

A one-stage process of esterification of BE with propionic acid results in betulin dipropionate, 3β,28-di-O-propionyl-lup-20(29)-lupene (BDP) (Figure 9A). Under appropriate conditions, treatment of crushed birch bark with propionic acid leads to both the extraction of BE and the esterification of BE to BDP. BDP is characterized by poor solubility in water, which limits its potential use in medicine [55]. The mechanochemical methods were used to improve the solubility and bioavailability of BDP. For this purpose, a powder composite of BDP with substances such as PEG, polyvinylpyrrolidone, or fumed silica was produced. A composite was also made in the form of a water-soluble film with BDP and arabinogalactan. The BDP and the obtained composites were evaluated against human lung adenocarcinoma cells and Ehrlich ascites carcinoma cells. The obtained results showed a decreased activity of the mechanochemically modified BDP in comparison to the initial compound, and a distinct advantage of the form of water-soluble films. This work demonstrates not only the advantages of water-soluble composites of BDP in the context of their anticancer activity, but also the possibility of using solutions that do not require complex and expensive synthesis methods [55].

### 4.8. 3-Substituted Derivatives of Betulin and Betulinic Aldehyde

The other derivatives with anticancer properties may be obtained by the modification of the C-3 position of the BE molecule. They were obtained by introducing alkyl, alkenyl, and alkynyl chains to the acyl group in the C-3 position of the BE or betulinic aldehyde molecule. The antiproliferative activity of the compounds was evaluated in vitro against five human cancer cell lines: biphenotypic B myelomonocytic leukemia (MV-4-11), lung (A549), prostate (Du-145), melanoma (Hs294T), and breast (MCF-7) cancer cells, and a normal human mammary gland (MCF-10A). 3-(2-butynoyl)betulin (Figure 9B) exhibited the highest anticancer activity against prostate cancer, melanoma, and breast cancer cell lines. Molecular docking studies were performed to investigate the binding mechanism of the 3-substituted derivatives of BE and betulinic aldehyde with Akt protein [56].

### 4.9. Betulin Sulfonamides

**Figure 10:**
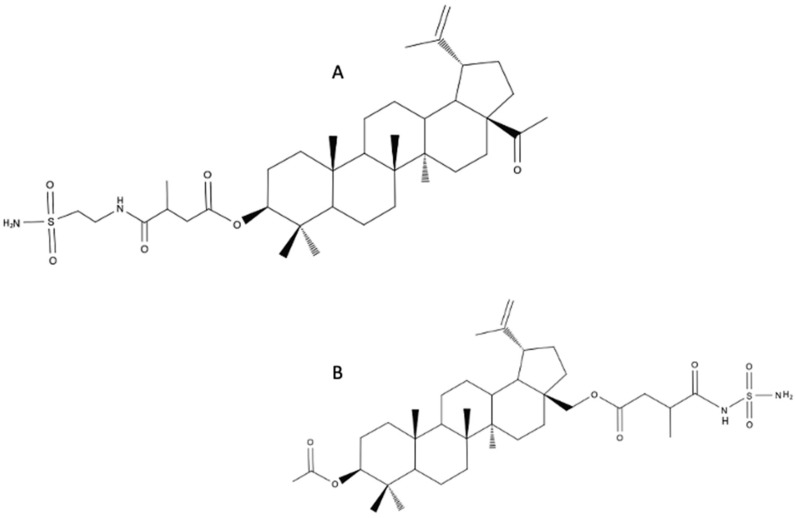
Chemical structure of (1R)-3a-(acetoxymethyl)-5a,5b,8,8,11a-pentamethyl-1-(prop-1-en-2-yl)icosahydro-1H-cyclopenta[a]chrysen-9-yl 3-methyl-4-oxo-4-(2-sulfamoylethylamino)butanoate (**A**) and (((1R)-9-acetoxy-5a,5b,8,8,11a-pentamethyl-1-(prop-1-en-2-yl)icosahydro-1H-cyclopenta[a]chrysen-3a-yl)methyl 3-methyl-4-oxo-4-(2-sulfamoylethylamino)butanoate) (**B**).

Betulin sulfonamides are considered agents of potential use in the treatment of breast cancer. Sulfonamides are known as carbonic anhydrase inhibitors. Excessive expression of carbonic anhydrase correlates with a poor prognosis in some types of cancer [57]. Two compounds: (1R)-3a-(acetoxymethyl)-5a,5b,8,8,11a-pentamethyl-1-(prop-1-en-2-yl)icosahydro-1H-cyclopenta[a]chrysen-9-yl 3-methyl-4-oxo-4-(2-sulfamoylethylamino)butanoate (Figure 10A) and (((1R)-9-acetoxy-5a,5b,8,8,11a-pentamethyl-1-(prop-1-en-2-yl)icosahydro-1H-cyclopenta[a]chrysen-3a-yl)methyl 3-methyl-4-oxo-4-(2-sulfamoylethylamino)butanoate) (Figure 10B) were investigated. The in vitro activity of betulin sulfonamides against breast cancer cell lines (HS578T, MDA-MB-231, BT-20, MCF-7, T47D, SKBR3) was higher than the activity of BA. Especially promising seems to be the results against the triple-negative breast cancer line [58].

### 4.10. Betulin Ester with L-2,4-Diaminobutyl Acid

The effect of BE ester with L-2,4-diaminobutyl acid on the stimulation of collagen synthesis in human fibroblasts was analyzed. Plants from the *Betulaceae* family are known to be used for skin regeneration and healing ulcers, but there have not been many studies dedicated to evaluating the effect of particular ingredients. The new compound, betulin-Dab-NH_2_ (Figure 9C), was compared with BE and BA. The new compound showed improved solubility in water. Incubation of a primary fibroblast in the presence of the tested compounds at a concentration of 6 μM revealed that betulin-dab-NH_2_ enhances collagen synthesis in fibroblasts 7.86 times more than BA and 6.31 times more than BE [59].

## 5. Delivery Systems

**Figure 11:**
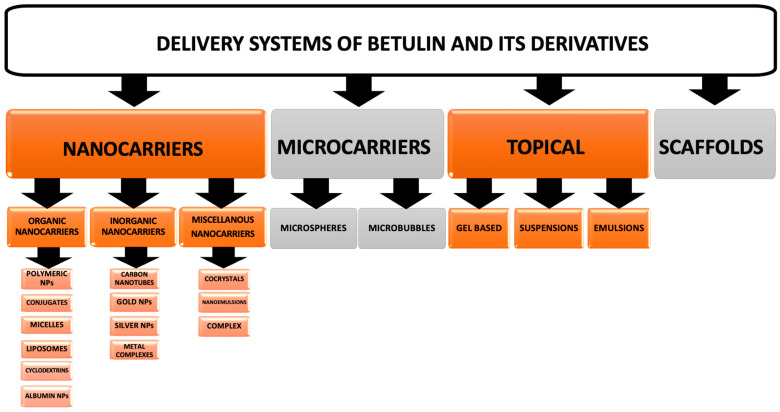
Different types of delivery systems designed for BE and its derivatives.

Drug delivery systems (DDSs) are used for the administration of an active agent into the body to achieve therapeutic efficacy [60]. The DDS enables the release of the active pharmaceutical ingredient to achieve a desired therapeutic response. Controlled drug delivery systems (CDDSs) have been developed to eliminate common limitations of conventional DDSs in terms of their poor aqueous solubility, poor drug selectivity, uncontrolled release, short period of bioavailability, high side effects, poor patient compliance profile in the case of chronic inflammatory disease treatment, premature metabolism, premature excretion, and poor bioavailability. There has been a significant evolution in CDDSs over the past two decades ranging from macro-scale and nano-scale to intelligent targeted delivery [61]. The development of innovative formulations like drug particle size design (nano- to millimeters, micro- to nano-encapsulated drug particles, and microchambers) that improve the water solubility and bioavailability of a drug is a significant aspect of current drug delivery technology [60]. The controlled release drug delivery system (CRDDS) refers to the release of the drug either rapidly or slowly. The CRDDS maintains a predictable predetermined or constant rate as well as reproducible pharmacokinetics, including a steady plasma drug level for a long period of time (single day to several month) with steady safety and efficacy besides improved patient compliance [60]. There has also been significant progress in the development of delivery systems of BE and its derivatives, involving nanocarriers and microparticles that are discussed below. The division of the described drug delivery systems is presented in Figure 11. The latest advancements in the field are presented in Table 1.

### 5.1. Nanocarriers

Nanoparticles (NPs) present great promise for improving DDSs, allowing for the targeted and controlled release of an active agent. This has the potential to enhance the efficacy and reduce the side effects of drug therapies. Encapsulating drugs within NPs protects them from degradation, delivers them directly to the target site, and releases them in a controlled manner, maximizing their therapeutic effects [62]. Nanocarriers are a broad class of DDS that involve polymeric NPs, mesoporous NPs, nanomaterials, carbon nanotubes, dendrimers, liposomes, metallic NPs, nanomedicine, and engineered nanomaterials (Figure 11).

The importance of nanocarriers has rapidly grown to treat certain diseases like cancer (e.g., brain, lung, and breast cancer), cardiovascular diseases, and many others. NPs can be engineered to have specific surface properties that allow them to selectively target diseased cells while avoiding healthy tissues, which can increase the efficacy and reduce the side effects of drugs. Additionally, NPs can be designed to release their cargo in a controlled manner, allowing for sustained drug release [63,64,65]. Below, examples of organic and inorganic NPs designed for the delivery of BE and its derivatives are presented.

#### 5.1.1. Organic Nanocarriers

Organic NPs have been widely explored for decades and contain many types of materials: liposomes, the first nano-scale drug approved for clinical application, and polymer-based NPs [65]. Polymeric nanoparticles show low toxicity, increase therapeutic effectiveness, improve drug penetration, control drug release, and enhance physical and chemical stability [66]. This wide group of drug carriers involves nanospheres, nanocapsules, polymeric micelles, dendrimers, etc.

##### Polymeric NPs

**Figure 12:**
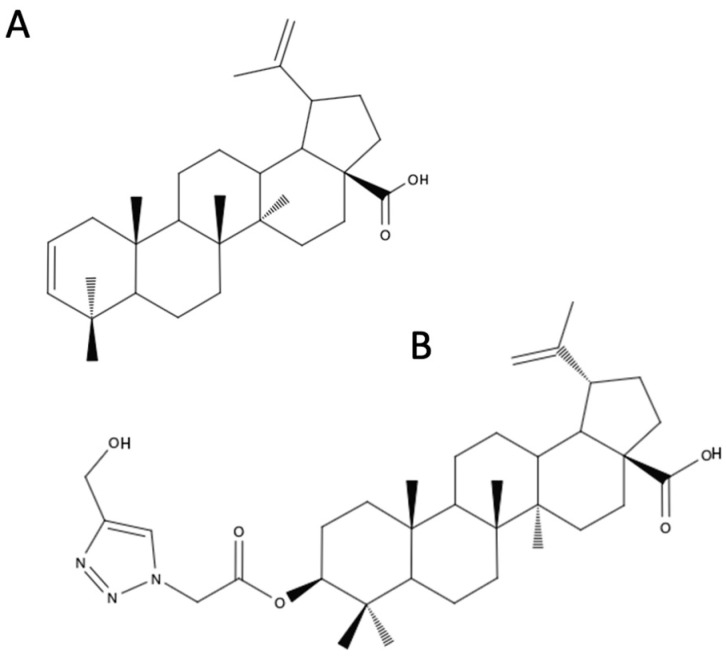
Chemical structure of 1-isopropenyl-5a,5b,8,8,11a-pentamethyl-1,2,3,4,5,5a,6,7,7a,8,11,11a,11b, 12,13,13b-octadecahydro cyclopenta[a]chrysene-3a-carboxylic acid (**A**), BA analogue (**B**).

One of the first attempts to create NPs with a BA derivative was made by Das et al. A derivative of BA (dBA) obtained from *Phytolacca americana* (1-isopropenyl-5a,5b,8,8,11a-pentamethyl-1,2,3,4,5,5a,6,7,7a,8,11,11a,11b, 12,13,13b-octadecahydro cyclopenta[a]chrysene-3a-carboxylic acid) (Figure 12A) was encapsulated into poly(lactide-co-glycolide) (PLGA) by the solvent displacement technique [67]. The mean diameter of the obtained NPs was around 110 nm [67]. The study demonstrated that both dBA and dBA loaded into NPs decreased the viability of lung cancer cells (A549) in a dose-dependent manner. The results showed that dBA and dBA loaded in NPs, directly targeted the mitochondrial oxidative phosphorylation system. However, a stronger effect was observed for dBA loaded in NPs, indicating that they may be a better candidate for the development of an anticancer drug for use against lung adenocarcinomas. The study also helped to understand the mechanism of action of dBA, its influence on mitochondria, and the process of directing cells to the apoptotic pathway [67]. The influence of the tested molecules on the increase in the intracellular concentration of Ca^2+^ released from the endoplasmic reticulum (ER), which is one of the apoptotic factors, has been shown. Furthermore, an increase in ROSs and a decrease in the rate of oxidative phosphorylation have been observed, indicating the potential impact of these factors on mitochondria [67]. Moreover, an in vivo study was conducted to determine the potential of dBA-loaded NPs for absorption in various tissues. It has been shown that dBA loaded in NPs is distributed to tissues faster than dBA, and it can cross the blood–brain barrier (BBB) [68].

A nanoformulation of a BA analogue (Figure 12B) based on PLGA was developed for the treatment of colorectal cancer. The BA derivative has a 1,2,3-triazole moiety attached to the C-3 hydroxy group of BA through a linker. The in vitro drug release from the formulation proceeded in a sustained manner according to the Higuchi model of kinetics. The designed nanoformulation was assessed for its cytotoxic potential in vitro in human colorectal cancer cells (HT-29). It was observed that apoptosis significantly increased after the exposition of cells to the BA derivative encapsulated in NPs compared to a free drug. The bioavailability and tumor specificity of nanoformulation were confirmed by in vivo biodistribution studies [69].

**Table 1:** Latest reports regarding nanoparticulate drug delivery systems containing betulin or its derivatives.

Active Compound	Effect	Mechanism of Action	Delivery System	Cell Line	Animal Model	Ref.
dBA	anticancer	apoptosis; target the mitochondrial oxidative phosphorylation system	polymeric NP	A549	-	[67]
BA analogue	anticancer (colon)	apoptosis	polymeric NP	HT-29	rats, mice	[69]
BA	anticancer (liver)	n/a	polymeric NP	HepG2	-	[70]
Betulinic amine	antioxidant for ischemic stroke	decreased BBB leakage, improved tight junction repair, and reduced brain edema	polymeric NP	-	rats, mice	[71]
BA	anticancer (anti-glioma)	therapeutic effects are mainly mediated by CB1/CB2 through suppression of the Akt/NFκB-p65 signaling	polymeric NP	U87, A172	mice	[72]
BA and gemcitabine	anticancer (pancreas)	apoptosis	polymeric NP	Panc1	mice	[73]
BA	hepatoprotective	reduce the degree of liver fibrosis	polymeric NP	-	rats	[74]
BA	anticancer (TNBC, larynx)	apoptosis	polymeric NP	HEp-2, MDA-MB-231		[38]
BA	anticancer (TNBC)	induction of DNA double-strand damage, induction of ROS accumulation, angiogenesis inhibition	micelle	MDA-MB-231	-	[75]
BE derivative	anticancer (TNBC, ovary)	apoptosis	micelle	HeLa, SK-BR-3	-	[76,77]
BA	anticancer (pancreatis)	apoptosis	conjugate	MIA, PaCa-2	-	[78]
BA	anticancer	n/a	liposome	A549, SW480	mice	[37]
BA	anticancer (liver, ovary)	n/a	liposome	HepG2, HeLa, U14	mice	[79]
BA	anticancer (liver)	n/a	liposome	HepG2	-	[80]
BA	anticancer (liver)	mitochondrial-related apoptosis	liposome	HepG2	-	[81]
BE	anticancer (melanoma)	apoptosis	cyclodextrin	B164A5	mice	[82]
BA and doxorubicin	anticancer (lungs)	apoptosis, induction of ROS accumulation	protein nanocarrier	A549	-	[83]
BE	antimicrobial (leishmaniasis)	n/a	carbon nanotubes	J774A.1 infected with *L. donovani*	-	[84]
BE	anticancer (melanoma)	apoptosis	gold nanocarrier	HaCaT, 1BR3, A375, B164A5	-	[85]
BE	anticancer antiangiogenic	n/a	nanoemulsion	-	mice, chick embryo CAM	[86]
BA	antiangiogenic	limiting the development of capillaries by modulating the activity of fibroblasts	nanoemulsion	-	chick embryo CAM	[87]
BE	anticancer antiangiogenic (breast)	apoptosis, inhibition of blood vessel development	nanosuspension	MDA-MB-231	-	[88]
BA	anticancer antioxidative (melanoma)	n/a	cocrystals	HaCaT, B164A5, B16F0	-	[89]
BA and Ceranib-2	anticancer (prostate)	induction of ROS	nanocarrier	PC-3	-	[90]
BE	anticancer antibacterial	effects on mitochondria	conjugate	HCT 116, MCF-7	-	[91]
BA	anticancer	mitochondrial-related apoptosis	conjugate	Caco-2, HeLa, MCF-7	-	[92]
BA	anticancer	apoptosis, release of NO	conjugate	B16F10	-	[93]
BA	anticancer (liver)	apoptosis, caspases inhibition	nanocarrier	MHCC97H, L02	-	[94]
BA	anticancer (cervix)	synergistic effect of photothermal therapy and the cytotoxic effect of the active compound.	liposome	143B, HeLa	mice	[95]
BA	anticancer (cervix)	synergistic effect of photothermal therapy and the cytotoxic effect of the active compound.	liposome	HeLa	mice	[96]
BA	anticancer (cervix)	synergistic effect of photothermal therapy and the cytotoxic effect of the active compound.	liposome	HeLa	mice	[97]
BA	anticancer antiangiogenic	synergistic effect of hyperthermal therapy and the cytotoxic effect of the active compound	liposome	MDA-MB-231, MCF-7	chick embryo CAM	[98]
BA	anticancer (breast)	n/a	cyclodextrin	4T1, MCF-7	mice	[36]
BE	anticancer	n/a	nanocarrier, microparticle	KB, HeLa, MCF-7, Hep-G2, A549, U87, HDF	-	[99,100]
BE	anticancer (melanoma)	apoptosis	silver nanocarrier	B165A5, B16Ova	mice	[101]
BA	antimicrobial	n/a	metal complexes	-	-	[102]

BA was also encapsulated in glycosylated zein (GZ) NPs (BA-GZ NPs) [70]. Zein is a natural, biocompatible, and biodegradable polymer widely used in the pharmaceutical, biomedical, and packaging fields because of its low water vapor permeability, antibacterial activity, and hydrophobicity [103]. The process of protein glycosylation improves the solubility, bioactivity, stability, and encapsulation efficiency of active ingredients. The efficiency of BA encapsulation in Zein NPs was lower than that of GZ NPs. The BA-GZ NPs showed antitumor activity against hepatoma carcinoma cells (HepG2) [70].

**Figure 13:**
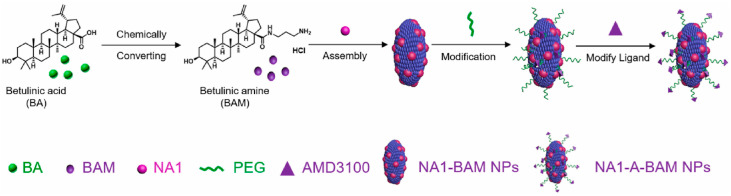
Schematic diagram of NA1-A-BAM NP synthesis [71].

The development of natural compound-derived NPs has been reported, which have a dual function, as a potent therapeutic agent for stroke treatment and an efficient carrier for drug delivery to the ischemic brain. Zhang et al. engineered BA NPs for preferential drug release in acidic ischemic tissue by chemically converting BA to betulinic amine (BAM) (Figure 13). The targeted drug delivery was obtained through surface conjugation of AMD3100, a CXCR4 antagonist. The resulting AMD3100-conjugated BAM NPs were used as a carrier for NA1, a fusion peptide that protects neurons from NMDA receptor-mediated neuronal damage (NA1-A-BAM NP). They cross the blood–brain barrier and target the release of the drug in the ischemic tissue [71]. It was also demonstrated that BA NPs may inhibit glioma cell proliferation and that the therapeutic effect of BA NPs is influenced by the presence of CB1/CB2 receptors on the surface of glioblastoma cells [72].

Biodegradable PLGA-mPEG polymeric nanocarriers were developed for the effective and simultaneous delivery of two drugs—hydrophilic (gemcitabine) and hydrophobic (BA) (GEM + BA NPs). The NPs were characterized by their spherical shape and size below 200 nm. The GEM + BA NPs exhibited significantly improved cytotoxicity in a pancreatic cancer cell line (Panc1) compared to native drugs or single drug-loaded NPs. The increase in cytotoxic activity was consistent with the cellular production of ROSs, which was mediated through increased cell apoptosis. Moreover, an in vivo study demonstrated the superior antitumor efficacy of the GEM + BA NPs compared to native drugs [73].

Covalently conjugated galactose is used to target the liver because it is recognized by asialoglycoprotein receptors, which are exclusively expressed in hepatocytes. Therefore, galactosylated chitosan (GC) was used to form the BA-loaded GC NPs (BA-GC NPs) for the treatment of liver fibrosis. It was shown that BA might be applied for the treatment of liver fibrosis because it reduces the pathological damage related to liver fibrosis and lowers the serum platelet-derived growth factor and serum hydroxyproline levels by activating autophagy [104]. The ionic cross-linking method was used to prepare the NPs. An in vitro study showed that BA is released from BA-GC NPs in two stages, with over 30% rapidly released during the first phase (0.5–12 h) from the NPs’ surface. In the second step, the remaining drug is released steadily over the next 12–96 h due to the gradual erosion and degradation of the NPs. The BA-GC NPs demonstrated good biocompatibility at the cellular level and a lack of any inflammatory reaction in mice. The in vivo study also exhibited a liver-targeting effect and reduced degree of liver injury in a mouse model of liver fibrosis BA-GC NPs [74].

Lactoferrin (Lf)–targeted PLGA NPs loaded with BA (LF-BA-PLGA NPs) were developed for the treatment of triple-negative breast cancer (TNBC) human larynx epidermoid carcinoma [38]. Lactoferrin (Lf) is an iron-binding glycoprotein that has a strong binding affinity with overexpressed transferrin receptors prevalent in breast cancer cells including TNBC [105,106]. Furthermore, Lf forms an important component of the immune system in humans and modulates cellular protein kinase and Fas signaling pathways [38]. The solvent diffusion method was used to prepare the LF-BA-PLGA NPs. The targeted NPs were exposed to two highly invasive and metastatic cell lines, HEp-2 and MDA-MB-231, and were shown to achieve cellular uptake and induce cancer cell death [38].

##### Micelles

Micelles have also been the subject of research among the nanocarriers used as a drug delivery system. Polymeric micelles have a core–shell structure formed by the self-assembly of amphiphilic block copolymers which can solubilize poorly water-soluble drugs. The loading of the drug into micelles allows the drug’s limitations to be reduced, such as hydrophobicity or poor bioavailability [107].

**Figure 14:**
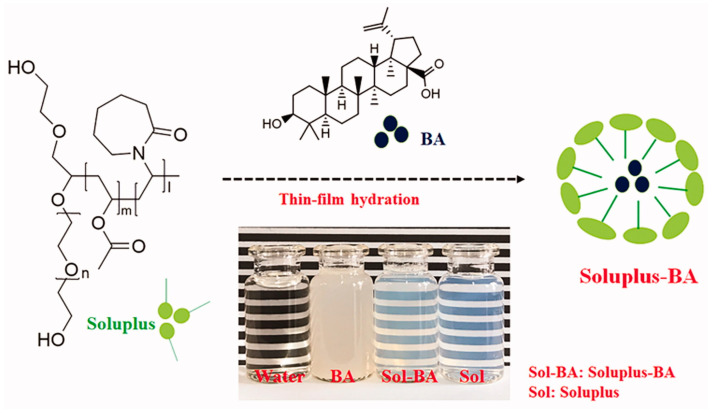
Schematic illustration of self-assembled Soluplus–BA micelles [75].

Polyvinyl caprolactam-polyvinyl acetate-polyethylene glycol (PVCL-PVA-PEG) graft copolymer (Soluplus) was used to form micelles encapsulated with BA by a thin-film dispersion method (Figure 14). Soluplus–BA micelles increased the inhibitory effect of BA in the breast cancer cell line (MDA-MB-231). The detailed analysis of the mechanism of action showed that Soluplus–BA induced DNA double-strand breaks and induced cell apoptosis by inducing ROS accumulation. Moreover, the BA delivered in micelles also caused the angiogenesis inhibitory effect by regulation of the HIF-1/VEGF-FAK signaling pathway [75].

Advantages of the micelles also include the ability to conjugate with targeting molecules via surface modification, thus achieving the specific targeting and reducing the nonspecific uptake by the reticuloendothelial system (RES) [108]. Folate drug delivery systems can target folic acid receptors (FARs) that are overexpressed in several human carcinomas, which can potentially maximize therapeutic efficacy while minimizing side effects. Overexpression of the FARs is observed in human carcinomas including breast, ovary, endometrium, kidney, lung, head and neck, brain, colon, and myeloid cancers, while being only minimally distributed in normal tissues [109]. Another improvement may also be using worm-like micelles that are also called filomicelles, which possess a long circulation time of up to one week in the bloodstream and display almost a twice higher drug loading capacity as compared to spherical micelles due to their larger core volume per carrier [110,111]. Filomicelles for the delivery of BE derivatives (30-diethoxyphosphoryloxy-28-O-propynoylbetulin) (Figure 3C) were developed from the combination of two polymers—poly(L-lactide)-Jeffamine-folic acid and poly(L-lactide)-poly(ethylene glycol). The study showed the influence of PLA block on the initial burst effect. Additionally, the length of the copolymeric blocks affected the micelles’ morphology. TEM analysis revealed that filomicelles were exclusively obtained from PLA-Jeff-FA and PLA_5500_PEG_5000_ or PLA_3000_PEG_2000_, whereas coexistence of filomicelles and spherical micelles was observed from PLA-Jeff-FA/PLA_2300_PEG_2000_. The in vitro cytotoxicity of BE derivative-loaded micelles against FAR-positive HeLa cells was confirmed [76]. Analysis of the micelles obtained from a combination of PLA-Jeff-FA with PLA_5000_-PEG_5000_, PLA-PEG-FA with PLA_5000_-PEG_5000_, or PLA-Jeff-FA with PLA_3000_-PEG_5000_ showed that the length of the hydrophobic block is the main factor that controls inter- and intramolecular interactions and, in consequence, the micelles’ properties, e.g., drug encapsulation efficiency and release rate. Despite differences in the micelles’ drug loading and release properties, all kinds of micelles provided release of E-29-diethoxyphosphoryl-28-O-propynoylbetulin (Figure 2E) for over 264 h. Moreover, all kinds of drug-loaded micelles exhibited toxicity to human breast cancer (SK-BR-3) cells at concentrations above 10 μM. The largest cytotoxic effect was observed for PLA_5000_PEG_5000_ + PLA-Jeff-FA micelles with an elongated shape (short filomicelles) [77].

##### Conjugates

Another approach for the design and synthesis of novel therapeutic agents is the conjugation of the skeleton molecule with a moiety that will increase the effectiveness and physicochemical parameters. BE and its derivatives contain functional groups in their skeleton that can be modified to affect their biological activity. Therefore, prodrugs from BE are developed by its conjugation with other molecules. BE covalently linked via an amide bond to PEG (PEG-BA) is more effective against pancreatic cancer cells compared to free BA. The PEG-BA was tested for its effect on cell death, immunomodulation, and chemoresistance-linked signaling pathways. The conjugate was significantly more toxic to prostatic adenocarcinoma cells (PCs) and cytotoxicity was confirmed by increased apoptosis. It inhibited the production of IL-6 and caused the dysregulation of crucial chemoresistance genes such as *WNT3A*, *TXNRD1*, *SLC2A1*, and *GATA3* [78].

##### Liposomes

Liposomes (LSs) are one of the most widely studied types of NPs, especially for their ability to encapsulate and deliver various therapeutic agents, including chemotherapy drugs, peptides, and nucleic acids [62]. LSs are lipid-based spherical vesicular systems composed of two hydrophilic layers between a lipophilic bilayer. The entrapment of both hydrophobic and hydrophilic drugs into liposomes is possible [65]. They not only protect drug molecules from degradation, but also provide controlled release of drugs, modification of biodistribution, targeted drug action, and increased drug bioavailability and solubility [112].

An in vivo study was performed to prove the activity of BA-encapsulated LSs on human lung (A549) and colon cancer (SW480) cell lines that were inoculated into female athymic nude Foxn1 mice. It was demonstrated that in mice with both types of tumors, it was possible to significantly inhibit tumor growth by intravenously administering LSs containing BA [37].

**Figure 15:**
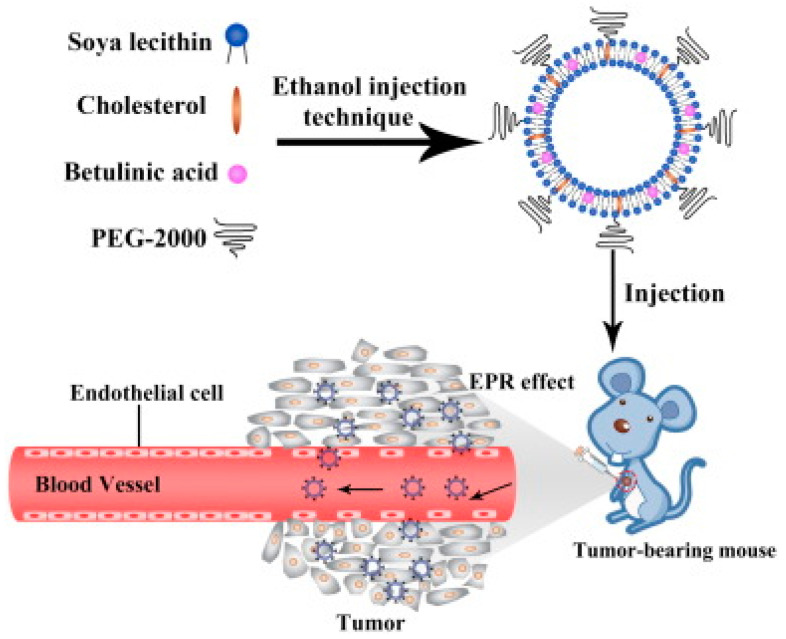
Schematic illustration of the synthetic route to the PEGylated BA liposome and cancer therapy [79].

However, the applications of conventional liposomes are still challenged by poor physical and chemical instabilities (aggregation or fusion, precipitation), low drug loading efficiency, and a relatively short blood circulation time. To increase its clinical acceptability, the surface of liposomes is modified with polyethylene glycol (PEG). PEGylated liposomes are regarded as stealth liposomes, which significantly extends their blood circulating time, decreases interaction with plasma proteins, and reduces uptake by the reticuloendothelial system [113]. PEGylated LSs containing BA with a size of 142 nm were reported (Figure 15). An in vitro drug release study showed that the PEGylated LSs were more stable and released BA more slowly than PEG-free LSs. Studies conducted on cervical cancer (HeLa) and liver cancer (HepG2) cell lines have confirmed the antitumor efficacy of PEGylated LSs containing BA. The antitumor activity of the prepared formulation was also confirmed in mice after the injection of U14 cells of cervical cancer [79].

The surface of LSs with encapsulated BA can also be modified with particles enabling a targeting effect, e.g., with folate. An enzymatic reaction was used to conjugate FA, PEG, and cholesterol, which is simpler than a chemical reaction and does not produce many by-products. The final LSs were characterized by a diameter of 220 nm. An in vitro study showed a reduced survival of the FR-positive human liver cancer cell (HepG2) treated with modified LSs compared to free BA and unmodified LSs, and no significant difference in survival was found for FR-negative human lung carcinoma cells (A549) [80].

Another group of molecules that can be used to modify the surface and thus the properties of LSs is glycolipid biosurfactants. They increase the stability of LSs, support the absorption of LSs through the cell membrane, and accelerate the fusion of the DNA-LS complex. Mannosylerythritol lipid-A was used for coating of LSs encapsulated with BA. The resulting LSs with a diameter of about 90 nm were tested on HepG2 cell lines and showed anticancer activity [81].

##### Other Organic NPs

Cyclodextrins (CDs) are a group of cyclic oligosaccharides produced from starch or its derivatives as a result of the activity of the bacterial enzyme cyclodextrin glycosyltransferase (CGTase). CDs are composed of at least six glucopyranose monomers linked via α-1,4-glycosidic bonds. Due to their specific structure, solubility in water, and ability to form complexes with other molecules, including compounds of plant origin, they are a promising object of scientific research. The benefits of incorporating drug molecules into complexes with CDs include increased stability and bioavailability, as well as a reduced risk of drug–drug and drug–excipient interactions, reduction in gastrointestinal irritation, and the ability to convert liquid drugs into a microcrystalline powder [114,115].

It has been demonstrated that cyclodextrin derivative octakis-[6-deoxy-6-(2-sulfanyl ethanesulfonate)]-γ-CD (GCDG) can form complexes with BE. The solubility of BE significantly increased as a result of cyclodextrin complexation, and the activity of the complex in vitro was significantly improved compared to the cyclodextrin derivative or free BE. The mass of the tumor and tumor dimensions in C57BL/6J mice inoculated with melanoma cells and treated with the BE complex were significantly reduced. The reduction in melanoma progression was also confirmed [82]. Further studies were conducted in order to determine the effect of the BA complex with GCDG on the proliferation and tumor development of non-metastatic and metastatic melanoma cells (B164A5). Slightly increased inhibitory activity on metastatic cells was observed compared to the non-metastatic mouse cell line. The BA-GCDG complex improved the antiproliferative activity, although this effect was not statistically significant [116].

NPs based on bovine serum albumin may be used for active targeting to cancer cells with the increased expression of albumin receptors. Nanoparticles of this type allow both the encapsulation of the drug molecule and the modification of its surface [117]. The albumin-based NPs have been developed for a combination therapy with doxorubicin and BA BSA-(Dox+BA). Viability assays of the BSA-(Dox+BA) against non-small-cell lung carcinoma (NSCLC) A549 cells demonstrated a higher synergistic cytotoxic activity than the two drugs with no carrier. Moreover, cellular internalization of BSA-(Dox+BA) and the accumulation of the Dox in the nucleus were confirmed. The mechanism of action of BSA-(Dox+BA) relied on S-phase cell cycle arrest, DNA damage, caspase cascade activation, and downregulation of epidermal growth factor receptor (EGFR) expression [83].

#### 5.1.2. Inorganic Nanocarriers

##### Carbon Nanotubes

Carbon nanotubes (CNTs) are particularly attractive because of their high surface area, high drug loading capacity, durability, and stability. Despite their many advantages, there are some concerns about their safety and toxicity and the fact that they are not biodegradable. However, further research may bring interesting results not only in the field of the DDS, but also in tissue engineering, diagnosis, and imaging [118]. There has also been study in using CNTs for BE delivery. For this purpose, BE was conjugated to the carboxylic acid chains on functionalized carbon nanotubes (f-CNTs) to obtain antileishmanial formulation (f-CNT-BE). Analysis of the cytotoxicity of BE, f-CNT, and f-CNT-BE on macrophage cells demonstrated their biocompatibility at cellular level. f-CNT-BET showed high antileishmanial activity with increased efficacy compared to BET as evidenced from an in vitro study [84].

One of the strategies for the improvement of the biocompatibility of the CNTs and to prevent rapid drug release was the development of a coating procedure by using one of four biomaterials—Tween 20, Tween 80, PEG, or chitosan. It was observed that all of the chemically coated samples released the drug in a slow, sustained, and prolonged manner compared to the uncoated systems. The in vitro cytotoxicity was dependent on the drug release profiles and the type of coating. Therefore, it was concluded that the initial burst, drug release pattern, and cytotoxicity could be well controlled by selection of the desired materials to adjust to particular therapeutic applications [119].

##### Gold Nanocarriers

The use of metallic NPs as a DDS and as diagnostic agents in cancer therapy has been studied. Gold NPs (Au NPs) have been shown to be biocompatible and highly effective in antitumor therapy compared to other metallic NPs. There are a number of properties that make them a suitable candidate for drug carrier use, including controlled synthesis, easy functionalization, passive/active targeting properties, and the ability to transport and protect loaded drugs. Two types of BE-conjugated Au NPs with and without the thiolated PEG of 50–70 nm were reported. The PEG coating improves the biocompatibility and circulation time. The in vitro evaluation of the tested samples indicated that BE-conjugated Au NPs showed a cytotoxic and apoptotic effect in an exposure time- and dose-dependent manner on selected cell lines (HaCaT, 1BR3, A375, B164A5) [85].

##### Silver Nanocarriers

Silver NPs functionalized with PEG have been developed for BE delivery. The resulting nanoparticles were validated for their anti-melanoma activity through both in vitro and in vivo studies. An in vitro study showed that within a specific concentration range, the NPs were cytotoxic to melanoma cells and have a pro-apoptotic mechanism of action. However, the NPs were neutral to normal cells [101].

##### Metal Complexes

The development of complexes of BA with metal ions, which would have higher antimicrobial activity and better antioxidant properties, was carried out through a two-step synthesis, in which the acid residue at the C-28 position was modified with hydrazine, and then, the resulting compound was reacted with the following metals: Fe, Cu, Zn, Sn, and Sb. The obtained complexes were tested on a panel of bacteria and fungi to confirm their ability to inhibit the growth of microbes. Unfortunately, the antioxidant properties were insignificant. However, the results represent the basis for continuing research with the use of BE derivatives [102].

#### 5.1.3. Complex/Hybrid and Miscellaneous Nanoparticulate Systems

An alternative DDS of BE or its derivatives is a nanoemulsion. A nanoemulsion, which is characterized by a droplet size between 20 and 200 nm, is used to increase the bioavailability of the drug, deliver it to the targeted site, or ensure prolonged circulation in the body [120]. An oil-in-water nanoemulsion with incorporated BE was made from flax-seed oil (BetNE) and evaluated for its antiangiogenic and anticancer properties. The Chick Embrio Chorioallantoic Membrane assay demonstrated the potential of BetNE to inhibit angiogenesis, and the study conducted on Balb/c mice with experimentally induced skin carcinoma confirmed the anticancer properties of BetNE [86]. Another nanoemulsion with antiangiogenic properties was obtained with BA [87]. The MDA-MB-231 breast cancer cell line was used to evaluate the activity of BE in the form of a nanosuspension. The performed tests provided information on the increased anticancer activity of such a formula in relation to BE. In addition, the obtained nanosuspension has the ability to inhibit angiogenesis, which was confirmed by the tail fin regeneration assay in the zebra fish model [88].

The possibility of increasing the solubility and dissolution rate of BE and BA by synthesis of cocrystals has been reported. Cocrystals are a combination of two compounds that form a crystalline structure based on non-covalent bonds. This allows their physical properties to be modified without affecting their biological activity [89]. Cocrystals of BE with dicarboxylic acid and BE with terephthalic acid have been obtained. The cocrystals showed improved solubility in comparison to BE. The use of a coformer, which has an effect on crystal nucleation and growth, ensures the inhibition of precipitation of hydrophobic drug molecules followed by crystallization of the substance [121,122]. The crystals from BA and vitamin C were evaluated for their anticancer activity on selected cell lines. The particular sensitivity of murine melanoma cells to the stimulation of BA cocrystals with vitamin C has been observed [89].

A nanocarrier was formed from a combination of gallic acid, polylactid acid, alginate, and Zn:MnO_2_ to combine BA activity with ceranib-2. Prostate cancer is associated with elevated levels of acid ceramidase, which is inhibited by ceranib-2. The developed carrier was stable and capable of encapsulating hydrophobic drugs. It can be used in therapy and diagnostics. The NPs with encapsulated BA and ceranib-2 exhibited cytotoxic activity against prostate cancer cells (PC-3) [90].

One of the strategies for the development of effective therapeutic agents is the conjugation of a native skeleton of BE or its derivative with triphenylphosphonium cation (TPP^+^) of low molecular weight, which promotes accumulation inside the cell’s mitochondria and improves the pharmacokinetic properties including solubility, bioavailability, and intermembrane transport as well as selectivity in targeting drugs for a specific purpose [2,123,124]. Several new molecular hybrids of BE were synthesized by covalent linkage of the alkyltriphenylphosphonium moiety to the parent skeleton of BE via the O(CO)CH_2_CR_2_COO linker [91].

The mitochondrial targeted delivery of BA was also obtained by the mitochondriotropic TPP^+^-functionalized epigallocatechin gallate (EGCG)-capped gold NPs (Au NPs). The EGCG-capped Au NPs were further functionalized with poly-L-lysine (PLL). EGCG has a strong affinity for the laminin receptor, which is overexpressed in some tumors (e.g., lung, thyroid, colon cancers). The average size of NPs did not exceed 150 nm. The modified NPs showed higher cytostatic activity than free BA against several cell lines (CaCo-2, HeLa, MCF-7, HEK293). Moreover, the targeted NPs caused significant mitochondrial membrane depolarization compared to non-targeted NPs and free BA. NPs based on PLL and PEG showed higher mitochondrial membrane depolarization than NPs based on PEG. The presented functionalized NPs need further studies, especially in vivo, to evaluate their clinical potential [92].

A successful attempt to form a BA hybrid by conjugation with the nitric oxide-releasing moiety was reported. It is known that nitric oxide has the properties of inducing apoptosis of tumor cells and sensitizing tumors to chemotherapy. The obtained derivative has been shown to have antiproliferative properties, especially against mouse melanoma cells (B16F10), compared to free BA or cisplatin. The newly formed derivative causes mitochondrial dysfunction in the G1 phase of the cell cycle [93].

**Figure 16:**
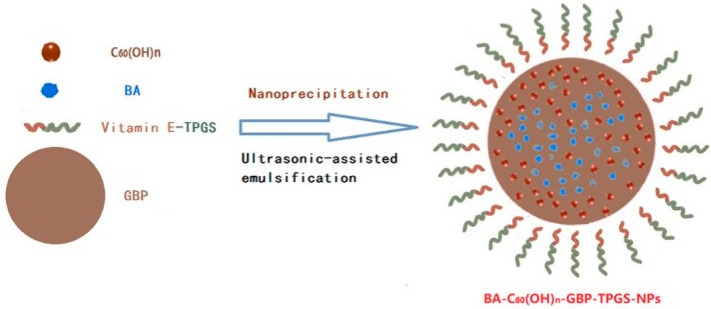
Scheme of preparation BA-C_60_(OH)_n_-GBP-TPGS NPs [94].

Nanoprecipitation with ultrasonic-assisted emulsification was used to form polyprenol lipid and vitamin E-TPGS hybrid NPs, which transport BA and low-substituted hydroxyl fullerenol (BA-C_60_(OH)_n_-GBP-TPGS NPs) (Figure 16) [94]. The goal of the engineered NPs was to limit the progression of hepatocellular carcinoma (MHCC97H). TPGS is a surfactant and a drug safety adjuvant with FDA approval. It inhibits the P-glycoprotein and also has a beneficial solubilizing effect, which improves the bioavailability of some drugs. The polyprenol found in *Ginkgo biloba* (GBP) leaves is a liposoluble ingredient that selectively increases the intracellular accumulation of chemotherapeutic drugs and the cytotoxins in MDR-related tumor cells [94]. Fullerenol (C_60_(OH)_n_) is a water-soluble original C_60_F, which is rich in hydroxyl groups and could efficiently inhibit the growth and metastasis of a transplanted malignant tumor. The formulation (BA-C_60_(OH)_n_-GBP-TPGS NPs) resulted in the controlled release of BA and C_60_(OH)_n_, which effectively inhibited the proliferation and migration of liver cancer cells in vitro [94]. The study also demonstrated an increase in Caspase-3, Caspase-8, and Caspase-9 expression in liver cancer cells, which correlated to BA concentration in NPs [94].

**Figure 17:**
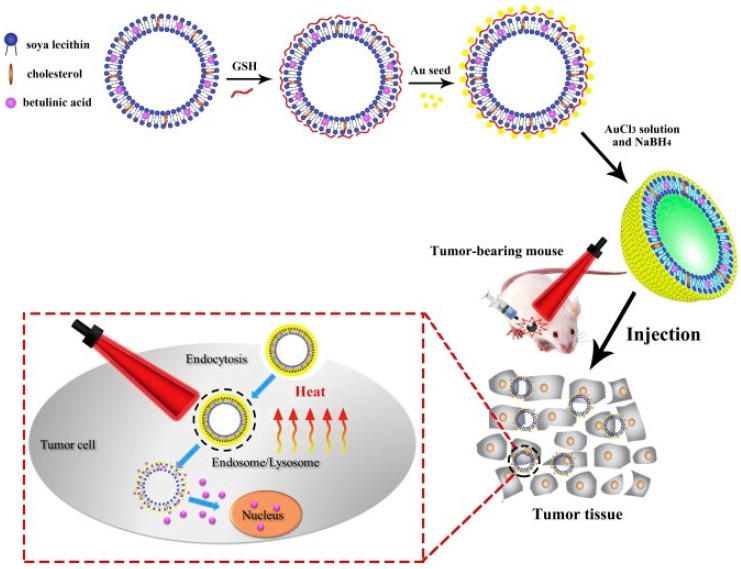
Schematic illustration of the synthesis route for the multifunctional Au NPs and the NIR laser irradiation-induced chemo-photothermal therapy in tumor-bearing mice [95].

A multifunctional drug delivery system has been developed from LSs containing BA with Au NPs for photothermal therapy (Figure 17). Glutathione was used as a bridge between Au NPs and the LS due to the formation of the Au-S bond. The LSs characterized an average diameter of 149 nm and showed a significant increase in bioavailability and cellular uptake under the influence of NIR light. The laser irradiation of the tumor, after administration of LSs, caused the enhanced release of BA, which inhibited tumor growth in U14 tumor-bearing mice [95]. Furthermore, surface modification of BA-containing LSs (gold-nanobranched coated LSs with BA or flower-like Au-Pd bimetallic nanocrystal-covered LSs with BA) enabled combined chemotherapy and phototherapy to be achieved [96,97].

The use of magnetic iron oxide nanoparticles (MIONPs) in drug delivery therapies is also an innovative approach. The encapsulation of MIONPs in LSs opens the possibility of remote delivery of the drug using an external magnetic field, which allows for a controlled transition of the magnetoliposomes (MLPs) from the gel state to the liquid state within the induced hyperthermia. The inclusion of MIONPs into LSs with encapsulated BA leads to the formation of MLPs with a diameter of 198 nm. BA-MLPs showed enhanced antitumor efficacy against breast adenocarcinoma (MDA-MB-231, MCF-7) cells, whereas their activity against non-tumorigenic breast epithelial cells (MCF 10A) was low [98].

Targeted NPs of BA composed of a combination of BA, cyclodextrin, gelatin, and TiO_2_ were obtained for anticancer application. The solubility of BA in water is enhanced by cyclodextrin. The gelatin is distinguished by the presence of modifiable functional groups and by the RGD sequence, which is recognizable by tumor cells. Due to its biocompatibility and antibacterial properties, the core of the nanoparticle was made of TiO_2_. The NPs were characterized by sizes from 200 to 300 nm. The NPs exhibited a significant inhibition effect on the murine breast cancer (4T1) cells in both in vitro and in vivo antitumor experiments [36].

The incorporation of BE derivatives into nanosystems improves their solubility and bioavailability. Betulin succinate is known to undergo polymerization with an acetic anhydride, resulting in the formation of polyDBB [99]. PolyDBB can be regarded as a form of polymer prodrug system due to its ability to bind to the polymer skeleton and release the active ingredient upon polymer degradation. In contrast to the unbound substance, it exhibits a diminished efficacy in vitro against normal cell lines. By creating a copolymer from polyDBB and PEG600, the degradation time can be shortened, which has a positive effect on the IC50 [99]. PolyDBB can be used to form microspheres or nanospheres. This allows for a more effective delivery of betulin succinate to the site of action. The size of the spheres can be adjusted by the homogenization speed, which allows for the creation of various systems for inhalation, intramuscular, implant, or intravenous administration [99].

### 5.2. Microparticles

Microparticulate systems such as microspheres, microcapsules, or any particle in a micrometer scale (usually of 1–1000 μm) are widely used as drug delivery systems because they offer higher therapeutic and diagnostic performance compared to conventional drug delivery forms [125]. The use of microspheres as a drug delivery system allows for encapsulation of the drug as well as its protection and controlled release. They are suitable for intravenous, oral, or inhalation administration. Microspheres can be made of a wide range of materials, but biodegradable polymers are the most common due to their ability to be eliminated from the body. Microspheres obtained from polyanhydrides of disuccinate BE and dicarboxylic derivatives of PEG (polyDBB PEG) were reported. They released the active substance—betulin disuccinate—as a result of hydrolysis under physiological conditions. The microspheres possessed suitable sizes (0.5–25 μm) and characteristics for use in inhalation systems, which was confirmed during an aerosolization study and makes them suitable candidates for use in the treatment of lung diseases [100]. It was observed that the morphological characteristics of the microspheres depended on the presence of a PEG segment within the structure of polyanhydrides. The porosity of the particles depended on the amount and molecular weight of the PEG used and also on the speed of homogenization. The most porous particles were obtained from polyanhydrides containing 20% wt. of PEG 600 by using a homogenization speed of 18,000 rpm [100].

An interesting example of the advanced delivery systems of BA may be microbubbles (MBs) [126]. Microbubbles are a new type of gas-filled vehicles for anticancer theranostics, with sizes of 1 to 10 μm, stabilized by a lipid layer or protein shell, and enhance the contrast by acting as resonators driven by the compressibility of their gaseous core [127,128,129]. The nanocomplexes of liposomes or nanostructured lipid carriers (NLC) charged with BA or another active agent are linked chemically by PEG2000 and avidin–biotin to MB, and are administered intravenously by sonoporation. Then, the MB-linked complexes deliver the nanocomplexes targeting the tumor cells [126].

### 5.3. Topical Formulations

#### 5.3.1. Gel-Based

There are some reports of a new type of hydrogel based on low molecular weight chitosan and betulin aldehyde. The combination of these two ingredients was possible due to the use of ultrasound and the substitution of part of the water with ethanol in the hydrogelation process. The resulting biomaterial exhibits a microporous structure and high stability, thereby presenting novel prospects for chitosan in medical and pharmaceutical fields [130].

Oleogels containing ZnO nanoparticles modified with BE and its derivatives were evaluated for their effectiveness in the treatment of burn wounds. An in vivo study showed that the developed formulation not only accelerated wound healing but also improved microcirculation around the wound and possessed antioxidant parameters. It is noteworthy to emphasize that this effect was primarily attributed to the presence of zinc ions. A slightly improved effect was noticed due to the synergistic impact of ZnO NPs and the triterpenes [131]. Further research has been conducted to produce a wound dressing based on bacterial cellulose combined with ZnO nanoparticles and BE diphosphate. Compared to the previously mentioned oleogel [131], this type of dressing does not reduce the wound surface as rapidly, but the healing process is more stable [132].

The possibility of making dressings based on sodium alginate was also evaluated. However, in this study, birch bark extract was used instead of one active ingredient. The obtained biomaterials were examined for antimicrobial properties, demonstrating dose-dependent efficacy, and in vivo tests confirmed the potency of the dressings and their effect on the rate of wound healing [133].

Episalvan, also known as Oleogel S-10, is a registered product in the form of oleogel containing birch bark extract. It was approved for marketing by the European Medical Agency in 2016. Results from phase 3 randomized clinical trials show an improvement in the superficial partial healing thickness of burn wounds compared to standardly used formulations. The study emphasizes the advantages of Episalvan, including its good tolerance and safety, as well as the need to develop standards of care for superficial partial thickness burn wounds [134].

Pârvănescu et al. prepared an oleogel containing BE and evaluated its capacity to regenerate damaged skin. The parameters of the obtained oleogel enabled its application, and the system was well tolerated by the skin. Additionally, the presence of BE had an impact on the increased rate of repair of discontinuities in the skin. Furthermore, the use of oleogel reduces erythema levels and enhances skin hydration [135].

The gel-based systems described above were intended for skin application. However, the use of the hydrogel as an injectable carrier for BA with anticancer properties has also been described. Dai et al. presented a complex system consisting of carboxymethylcellulose–BA as a prodrug, which forms self-assembled NPs with hydroxycamptothecine and in combination with α-cyclodextrin creates a thermosensitive hydrogel. In vitro studies demonstrated an increase in drug release with the increase in temperature. Cytotoxic activity was confirmed both in vitro with the LLC lung cancer line and in vivo [136].

#### 5.3.2. Suspensions

Aqueous suspensions of BE derivatives are being used to create a film with therapeutic properties. Betulin diacetate, like other BE derivatives, is poorly soluble in water. Malyar et al. used the ability to form complexes between betulin diacetate and arabinogalactan using microwave heating. Compared to traditional methods, 10 min of microwave heating shortens the time needed to obtain the film by several times. Furthermore, films obtained in this manner retain their cytotoxic properties, which was confirmed by in vitro tests on Ehrlich ascites tumor cell lines. It is worth mentioning that the mechanism of cell death caused by films obtained using microwaves was predominantly apoptotic, compared to the dominance of necrotic mechanisms caused by films obtained using classical methods [137].

### 5.4. Emulsions

Birch bark extract has also been used in aesthetic medicine for its skin-regenerating properties. The emulsion known as Imlan creme pur is comprised of 50% water, 45.5% jojoba oil, and 4.5% birch bark extract (containing 80% of BE). In studies on skin regeneration following laser skin resurfacing of the face by CO_2_ laser ablation, the emulsion was used as an example of an open approach to wound treatment, while a hydrocolloid dressing and gauze were used as examples of a closed approach to wound treatment. Studies have demonstrated the beneficial effects of emulsions containing birch bark extract in this type of aesthetic medicine procedure [138].

Adepoju et al. evaluated the emulsion with BE to assess its ability to regenerate cells of the pancreas and its potential for the treatment of type 2 diabetes. Intragastric administration of an emulsion containing BE to rats not only decreased the concentration of glucose and glycated hemoglobin, but also reduced biochemical parameters indicating organ damage, i.e., pancreas, kidney, and liver during type 2 diabetes [139].

An example worth considering for increasing the bioavailability of BE is the preparation of Pickering emulsion. In such an example of a w/o emulsion, the stabilizer is composed of solid particles, such as BE. During digestion, the oily continuous phase undergoes decomposition into free fatty acids, resulting in micelles that solubilize the BE present in the emulsion. Depending on its concentration in the emulsion, an increase in the availability of BE was demonstrated. The Pickering emulsion has a greater surface area than the BE dispersed in oil, resulting in a higher bioavailability of the substance. These observations provide the basis for the development of an emulsion-based drug delivery system for BE [140].

### 5.5. Scaffolds

Apart from nanoparticles, microparticles, emulsions, and formulations intended for topical administration, scaffolds containing a BE derivative, produced by means of an electrospinning method, have also been developed [141]. Electrospinning technology can be used to fabricate fibrous materials with various chemical compositions, macro- and microstructures, and morphologies [142]. The principle of electrospinning technology is that a high voltage electrostatic field transports the polymer solution to the spinning needle, where it is stretched and polished by the electric field force to produce nanofibers at the collection end [143]. During the past decades, electrospinning has been found to have numerous biomedical applications in tissue engineering, sensing, and drug delivery systems as well as industrial applications such as in textile industries [144].

Polyolefin fibrous mats (ca. 400 nm diameter) have been produced via the electrospinning technique to evaluate the antimicrobial potential of BE-containing polymers [141]. Firstly, BE was converted into two kinds of α,ω-diene derivatives with different methylene spacer lengths between the olefin and the ester group via an esterification reaction. Polyolefins were subsequently made by acyclic diene metathesis (ADMET) polymerization of betulin-based α,ω-diene. Antimicrobial activities of the prepared mats against *E. coli*, *S. aureus*, and *C. albicans* were up to 63.5%, 89.7%, and 86.7%, respectively, depending on the composition of polymers. Thus, the results evidenced that the developed mats provided antibacterial properties, and a sterile and safe environment for biomedical engineering applications [141].

The formation of Betulin/Methyl-β-cyclodextrin (Betulin/MβCD) inclusion complex nanofibers (Betulin/MβCD-IC-NF) was reported by the methyl-beta cyclodextrin (MβCD) inclusion of BE and electrospinning techniques. BE served as the guest molecule and MβCD acted as the main molecule. The solubility of BE increased with the increase in MβCD concentration. Moreover, its thermal stability, and antibacterial and antioxidant activity were improved [145].

There have also been some examples of the successful preparation of an electrospinning scaffold containing birch bark extract, which can be used for wound healing [145,146,147].

## 6. Conclusions and Future Directions

The diverse biological activity of natural BE has been confirmed by numerous research articles. However, despite the abundance of BE and well-developed isolation methods from plant material as well as many studies confirming its very good biological properties, its use as a potential therapeutic agent is limited due to its low bioavailability, high hydrophobicity, and insufficient intracellular accumulation [91]. Therefore, various derivatives of BE have been obtained with a broad spectrum of bioactivity in terms of their anticancer, antimalarial, antibacterial, antiviral, anti-inflammatory, or hepatoprotective properties [91]. The novel derivatives have been obtained mainly by modification of the C-3, C-28, or C-30 positions of the parent molecule (Figure 1). As a result, a series of novel derivatives of BE and BA has been obtained, involving acetylenic, phosphates and phosphonates, triazole hybrids of BE and BA, betulin-1,4-quinone hybrids, triphenylphosphonium analogues of BE and BA, betulin dipropionate, betulin sulfonamides, betulin ester with L-2,4-diaminobutyl acid, 3-substituted derivatives of BE, and betulinic aldehyde (Figure 2, Figure 3, Figure 4, Figure 5, Figure 6, Figure 7, Figure 8, Figure 9, Figure 10 and Figure 12) [42,43,44,45,46,47,48,49,50,51,52,53,54,55,56,57,58,59].

Another direction is also the development of delivery systems for BE and its derivatives that may improve the problems related with high hydrophobicity, low solubility, and poor bioavailability. Thus far, it is mostly NPs that have been considered for the delivery of BE and its derivatives, including organic NPs (e.g., micelles, conjugates, liposomes, cyclodextrins, protein NPs), inorganic (carbon nanotubes, gold NPs, silver NPs), and complex/hybrid and miscellaneous nanoparticulate systems (Figure 11, Table 1). The only examples of the use of microparticles are microspheres obtained from polyanhydrides of disuccinate BE and dicarboxylic derivatives of PEG [99] and microbubbles [126]. However, there are also some examples of formulations for topical administration, emulsions, and scaffolds. Importantly, all the studied delivery systems show an improvement in biological effectiveness compared to the free drug. Recent research also indicates a move towards developing targeted delivery systems with folate or lactoferrin [38,77,80]. Moreover, novel hybrid nanoparticles with BE derivatives have been studied. This approach aims to create the best possible nanocarrier using a polymer composition with the addition of metals, but also the possibility of encapsulating a BE derivative with a drug with a different action profile to increase the anticancer activity of the resulting product. The multidirectional potential of these compounds, as well as the rapid development of nanotechnology, provide compelling reasons for exploring novel approaches for the development of both single and multicomponent systems. The combination of BE-type compounds with metal ions, vitamins, or other types of drugs offers opportunities for the development of new therapies. Research has also attempted to clarify the mechanism of action of individual derivatives. Understanding the interaction between the active molecular component and the therapeutic target will facilitate the development of more effective therapeutic systems [83,85,92,94,95,148,149]. Further progress in these fields is expected in the future.

## Data Availability

Not applicable.

## Abbreviations

1BR3	human fibroblast cell line
A375	human malignant melanoma cell line
A431	human epidermoid carcinoma cell line
A549	human lung carcinoma cell line
ABCA1	ATP-binding cassette subfamily A member 1
ABCG	ATP-binding cassette subfamily G
ADAMTS5	a disintegrin and metalloprotease with thrombospondin type I motifs
ADMET	acyclic diene metathesis
AhR	aryl hydrocarbon receptor
Akt protein	protein kinase B
ALT	alanine transaminase
AMD3100	plerixafor; small-molecule inhibitor of CXCR4
ARNT	aryl hydrocarbon receptor nuclear translocator
AST	aspartate aminotransferase
Au	gold
B164A5	murine melanoma cell line
B16F10	murine melanoma cell line
B16Ova	murine melanoma cell line
BA	betulinic acid
Bak	Bcl-2 homologous antagonist/killer
Balb3T3	fibroblast cell line derived from a BALB/c mouse strain
BAM	betulinic amine
BAX	pro-apoptotic gene/protein
BBB	blood–brain barrier
BCL-2	anti-apoptotic gene/protein
Bcl2	B-cell lymphoma 2 protein
BDP	betulin dipropionate
BE	betulin
BetNE	nanoemulsion with incorporated BE
BoA	betulonic acid
BSA	bovine serum albumin
BT-20	human breast cancer cell line
C32	melanoma cell line
C57BL/6J	a strain of laboratory mouse
C6	rat glioma cell line
CaCo-2	human epithelial colorectal adenocarcinoma cell line
CB1	cannabinoid receptor type 1
CB2	cannabinoid receptor type 2
CCRF/CEM	Caucasian cell line derived from a patient with and continuous Epstein–Barr virus (EBV)-transformed lymphoblastoid cell line derived from a patient with acute lymphoblastic leukemia
CD	cyclodextrin
CDDS	controlled drug delivery system
CGTase	cyclodextrin glycosyltransferase
CK	creatine kinase
CNT	carbon nanotube
CO_2_	carbon dioxide
COX2	cyclooxygenase-2
CRDDS	controlled release drug delivery system
Cu	cooper
CXCR4	C-X-C chemokine receptor type 4
Dab	L-2,4-diaminobutyl acid
dBA	a derivative of BA
DDS	drug delivery system
DNA	deoxyribonucleic acid
Dox	doxorubicin
Du-145	human prostate cancer cell line
E-TPGS	D-α-tocopheryl polyethylene glycol 1000 succinate
ECBO	bovine enterovirus strain
EGCG	epigallocatechin gallate
EGFR	epidermal growth factor receptor
EPR	enhanced permeability and retention
ER	endoplasmic reticulum
ERK1/2	extracellular signal-regulated kinase 1 and 2
f-CNTs	functionalized carbon nanotubes
FAD	flavin adenine dinucleotide
FAK	focal adhesion kinase
FARs	folic acid receptors
FAS	fatty acid synthase
FDA	Food and Drug Administration
Fe	iron
GATA3	GATA transcription factor 3
GBP	polyprenol found in ginkgo biloba
GC	galactosylated chitosan
GCDG	octakis-[6-deoxy-6-(2-sulfanyl ethanesulfonate)]-γ-CD
GEM	gemcitabine
GPx	glutathione peroxidase
GR	glutathione reductase
GSH	glutathione
GZ	glycosylated zein
HaCaT	spontaneously transformed immortalized human keratinocyte cell line
HBA	23-hydroxybetulinic acid
HEK293	spontaneously transformed immortalized human keratinocyte cell line
HeLa	cervical cancer cell line
HepG2	human liver cancer cell line
HIV	human immunodeficiency virus
HL-60	human promyelocytic leukemia cell line
Hs294T	human melanoma cell line
HS578T	human breast cancer cell line
IC50	half maximal inhibitory concentration
IL-1 β	interleukin 1 β
IL-18	interleukin 18
IL-6	interleukin-6
iNOS	inducible nitric oxide synthase
Jeff	jeffamine
Keap1	Kelch-like ECH-associated protein 1
LCR-4	bovine enterovirus strain LCR-4
LDH	lactate dehydrogenase
Lf	lactoferrin
LLC	large cell carcinoma
LPL	lipoprotein lipase
LS	liposome
MBs	microbubbles
MβCD	methyl-β-cyclodextrin
MCF 10A	human mammary epithelial cell line
MCF-7	human mammary epithelial cell line
MDA	malondialdehyde
MDA-MB-231	human breast cancer cell line
MDR	multidrug resistance
MHCC97H	human hepatocellular carcinoma cell line
MIONPs	magnetic iron oxide nanoparticles
MLPs	magnetoliposomes
MMP13	matrix metalloproteinase-13
MV-4-11	human leukemia cell line
NA1	fusion peptide
NAD[P]H	nicotinamide adenine dinucleotide phosphate
NF	nanofiber
NIR	near-infrared
NLCs	nanostructured lipid carriers
NMDA	N-methyl-D-aspartate
NO	nitric oxide
NPs	nanoparticles
NQO1	NAD[P]H-quinone oxidoreductase
NSCLC	non-small cell lung cancer.
p-p38	phosphorylated p38 mitogen-activated protein kinase
p21	cyclin-dependent kinase inhibitor 1A
p38 α	p38 Mitogen-Activated Protein Kinase α
P388	murine leukemia cell line
p53	a tumor suppressor protein encoded by the TP53 gene
Panc1	human pancreatic cancer cell line
PC	prostate cancer
PC-3	human prostate cancer cell line
Pd	palladium
PEG	polyethylene glycol
pERK	phosphorylated extracellular signal-regulated kinase
pJNK	phosphorylated c-jun N-terminal kinase
PLA	polylactic acid
PLGA	poly(lactic-co-glycolic acid)
PLL	poly-L-lysine
polyDBB	polyanhydride of betulin disuccinate
PPARγ	peroxisome proliferator-activated receptor gamma
PVCL	polyvinyl chloride
PVA	polyvinyl alcohol
RES	reticuloendothelial system
RGD	arginine-glycine-aspartic acid
ROS	reactive oxygen species
RPMI-7951	human melanoma cell line
Sb	antimony
SGC7901	human gastric cancer cell line
SK-BR-3	human breast cancer cell line
SLC2A1	solute carrier family 2 member 1
Sn	tin
SNB-19	human glioblastoma cell line
SOD	superoxide dismutase
Sol	soluplus
SREBP 1c	sterol regulatory element-binding protein 1 c
SW480	human colon cancer cell line
SW707	human colon cancer cell line
T47D	human breast cancer cell line
T98G	human glioblastoma cell line
TEM	transmission electron microscopy
TiO_2_	titanium dioxide
TLR-4	toll-like receptor 4
TNBC	triple-negative breast cancer
TNF-α	the inflammatory cytokine tumor necrosis factor α
TP53	tumor protein p53
TPP+	tetraphenylphosphonium cation
TXNRD1	thioredoxin reductase 1
U14	murine cervical cancer cell line
VEGF	vascular endothelial growth factor
WNT3A	wingless-type MMTV integration site family member 3A
Zn	zinc
ZnO	zinc oxide
